# The chimeric GaaR-XlnR transcription factor induces pectinolytic activities in the presence of D-xylose in *Aspergillus niger*

**DOI:** 10.1007/s00253-021-11428-2

**Published:** 2021-07-08

**Authors:** Roland S. Kun, Sandra Garrigues, Marcos Di Falco, Adrian Tsang, Ronald P. de Vries

**Affiliations:** 1 Fungal Physiology, Westerdijk Fungal Biodiversity Institute & Fungal Molecular Physiology, Utrecht University, Uppsalalaan 8, 3584 CT Utrecht, The Netherlands; 2 Centre for Structural and Functional Genomics, Concordia University, 7141 Sherbrooke Street West, Montreal, Quebec, H4B 1R6 Canada

**Keywords:** *Aspergillus niger*, Chimeric transcription factor, CRISPR/Cas9, Pectinases, D-xylose

## Abstract

**Abstract:**

*Aspergillus niger* is a filamentous fungus well known for its ability to produce a wide variety of pectinolytic enzymes, which have many applications in the industry. The transcriptional activator GaaR is induced by 2-keto-3-deoxy-L-galactonate, a compound derived from D-galacturonic acid, and plays a major role in the regulation of pectinolytic genes. The requirement for inducer molecules can be a limiting factor for the production of enzymes. Therefore, the generation of chimeric transcription factors able to activate the expression of pectinolytic genes by using underutilized agricultural residues would be highly valuable for industrial applications. In this study, we used the CRISPR/Cas9 system to generate three chimeric GaaR-XlnR transcription factors expressed by the *xlnR* promoter by swapping the N-terminal region of the xylanolytic regulator XlnR to that of the GaaR in *A. niger*. As a test case, we constructed a P*pgaX*-*hph* reporter strain to evaluate the alteration of transcription factor specificity in the chimeric mutants. Our results showed that the chimeric GaaR-XlnR transcription factor was induced in the presence of D-xylose. Additionally, we generated a constitutively active GaaR-XlnR V756F version of the most efficient chimeric transcription factor to better assess its activity. Proteomics analysis confirmed the production of several pectinolytic enzymes by Δ*gaaR* mutants carrying the chimeric transcription factor. This correlates with the improved release of D-galacturonic acid from pectin by the GaaR-XlnR V756F mutant, as well as by the increased L-arabinose release from the pectin side chains by both chimeric mutants under inducing condition, which is required for efficient degradation of pectin.

**Key points:**

• *Chimeric transcription factors were generated by on-site mutations using CRISPR/Cas9.*

• *PpgaX-hph reporter strain allowed for the screening of functional GaaR-XlnR mutants.*

• *Chimeric GaaR-XlnR induced pectinolytic activities in the presence of D-xylose.*

**Supplementary Information:**

The online version contains supplementary material available at 10.1007/s00253-021-11428-2.

## Introduction

Filamentous fungi are efficient degraders of plant biomass, ensuring an essential role in the global carbon cycle. This is associated with their ability to produce and secrete large amounts of extracellular Carbohydrate Active enZymes (CAZymes, www.cazy.org) (Lombard et al. 2014), which have a variety of applications in different industrial fields, including food and feed, pulp and paper, or textile industries (Mäkelä et al. 2014).

Fungal Zn_2_Cys_6_ transcriptional activators play a key role in the regulation of enzyme production by activating the expression of genes encoding for enzymes required for the degradation of the substrates found in the environment. However, these transcription factors need to be activated to express their target genes (Whitmarsh and Davis 2000). Thus, the availability of inducing compounds may be a limiting factor in industrial production processes.

Pectinases have a broad application in food industry and are mainly used for juice clarification or in the production of jams, wine, coffee, and tea (de Vries et al. 2020). *Aspergillus niger* has a long history of safe application and is often used in industry for the production of valuable metabolites and enzymes (Cairns et al. 2018). In particular, this fungus is well known to possess a large array of genes encoding pectinases and accessory enzymes (Martens-Uzunova and Schaap 2009). A key transcription factor in the regulation of pectinase production is GaaR (Alazi et al. 2016), which is activated by its physiological inducer 2-keto-3-deoxy-L-galactonate, an intermediate compound in the pathway of D-galacturonic acid catabolism (Alazi et al. 2017), and has been shown to play a major role in the regulation of pectin degradation (Kowalczyk et al. 2017b).

The alteration of transcription factor specificity through the generation of chimeric transcription factors has been reported more than 30 years ago in yeasts (Corton 1989; Marmorstein and Harrison 1994; Witte and Dickson 1990). More recently, artificial transcription factors have been constructed in filamentous fungi to enhance the production of cellulases (Gao et al. 2017; Han et al. 2020; Zhang et al. 2016, 2017, 2018a, 2018b) or amylases (Yamashita et al. 2021). However, the application of this technique to facilitate or enhance pectinase production has not yet been reported.

The xylanolytic transcription factor XlnR was the first (hemi-)cellulolytic transcription factor described in *A. niger* (van Peij et al. 1998b), and it is the most studied transcriptional activator involved in the regulation of plant biomass degradation. It is involved in the colonization of plant biomass and in the degradation of its components such as xylan and cellulose (Kowalczyk et al. 2017a; van Peij et al. 1998a, 1998b). Moreover, D-xylose has been shown to activate XlnR (van Peij et al. 1998b), making this transcription factor a suitable candidate for the generation of D-xylose-inducible chimeric transcription factors. The generation of a GaaR-specific chimeric transcription factor that could be induced when cultivated on (hemi-)cellulose-rich agricultural waste materials would be a suitable approach for the industry, due to the high abundance of these substrates in nature.

In this study, we used CRIPR/Cas9 genome editing (Jinek et al. 2012; Song et al. 2018) to generate a chimeric GaaR-XlnR transcription factor through on-site modification of the endogenous *xlnR*. As a test case, we generated a hygromycin B-based reporter strain suitable for simple identification of functional chimeric transcription factor constructs. We showed that the chimeric GaaR-XlnR transcription factor *A. niger* mutant was able to secrete enzymes required for the degradation of pectin when growing on pectin supplemented with D-xylose as inducer compound.

## Materials and methods

### Strains, media, and growth conditions

Plasmids used in this study were propagated in *Escherichia coli* DH5α, which was grown in Luria-Bertani (LB) medium (Bertani 1951) supplemented with 50 μg mL^−1^ ampicillin (Sigma-Aldrich, St. Louis, MO, USA). The fungal strains used in this study were derived from *A. niger* CBS 138852 (*cspA1*, *pyrA*^*−*^, *kusA*::*amdS*) (Meyer et al. 2007), which was obtained from the Westerdijk Fungal Biodiversity Institute culture collection (Utrecht, the Netherlands). All strains generated in this study were deposited at the culture collection of Westerdijk Fungal Biodiversity Institute under accession numbers indicated in Supplemental Table S1. All fungal strains were maintained by growing at 30°C on *Aspergillus* minimal medium (MM) or complete medium (CM) (de Vries et al. 2004) supplemented with 1% D-glucose and 1.22 g L^−1^ uridine (Sigma-Aldrich, St. Louis, MO, USA).

Growth profiles were performed using *Aspergillus* MM with the addition of 25 mM D-glucose, D-galacturonic acid, or D-xylose (Sigma-Aldrich, St. Louis, MO, USA) or 1% beechwood xylan, cellulose, xyloglucan, or apple/citrus pectin. All media were supplemented with 1.22 g L^−1^ uridine. For antibiotic resistance tests, the media were supplemented with 10–25 μg mL^−1^ hygromycin B (InvivoGen, San Diego, CA, USA). All growth profile plates were inoculated with 1000 freshly harvested spores and performed in duplicates and were incubated at 30°C for up to 14 days. Growth was evaluated by visual inspection, and pictures were taken at multiple time points.

For liquid cultures, freshly harvested conidia were pre-grown in 250 mL CM containing 2% D-fructose (Sigma-Aldrich, St. Louis, MO, USA) and 1.22 g L^−1^ uridine for 16 h at 30°C in a rotary shaker at 250 rpm. After 16 h incubation, mycelia were harvested by filtration through sterile cheesecloth, rinsed with MM, and approximately 2.5 g (wet weight) mycelium was transferred in triplicates into 50 mL MM containing 2% wheat bran, 1% D-xylose, 1% citrus pectin (CP), or 1% citrus pectin supplemented with 0.075% (5 mM) D-xylose (CPX). Supernatant samples were taken after 24 h incubation at 30°C in a rotary shaker at 250 rpm. The samples were centrifuged (10 min, 3220 × *g*, 4°C), and cell-free supernatant samples were stored at −20°C until further processing.

### Construction of mutant strains

CRISPR/Cas9 genome editing was performed using the ANEp8-Cas9-*pyrG* plasmid, which contains the *pyrG* gene as selection marker (Song et al. 2018). The guide RNA (gRNA) sequences were selected by using the Geneious 11.1.4 software (https://www.geneious.com) based on the methodology described by Doench et al. (2014). Repair templates, which include ~ 750–1000 bp of the 5′ and 3′ flanking regions of the target sequences, were amplified and fused together using fusion-PCR and were used to repair the target sequence cleaved by the Cas9 nuclease.

The construction of CRISPR/Cas9 plasmids, generation of *A. niger* protoplasts, transformation, and colony purification of putative mutant strains was performed as previously described (Kun et al. 2020). The P*pgaX*-*hph* reporter strain CBS 147359 was generated by replacing the exopolygalacturonase X (*pgaX*) ORF (open reading frame) with the hygromycin-B-phosphotransferase (*hph*) ORF originated from *E. coli* (Kaster et al. 1983) in the *A. niger* CBS 138852 background strain. The mutants carrying D-xylose-inducible chimeric GaaR-XlnR constructs were generated by replacing the N-terminal region of XlnR with that of the GaaR in the *A. niger* CBS 138852, CBS 147359 (CBS 138852 P*pgaX*-*hph*), and CBS 146901 (CBS 138852 Δ*gaaR*) background strains. The constitutively active form of chimeric GaaR-XlnR V756F (corresponding to amino acid mutation V732F in the chimeric sequence) has been generated by simultaneous replacement of XlnR DNA-binding domain and insertion of a point mutation via a single-stranded oligonucleotide in the C-terminal region of XlnR as described before (Kun et al. 2020).

The generated mutant strains have been confirmed by diagnostic PCR, through the amplification of the target gene region and/or Sanger sequencing (Macrogen Europe, Amsterdam, the Netherlands) (data not shown). For each individual mutation, one candidate was selected for subsequent phenotypic analysis. All primers used in this study were ordered from Integrated DNA Technologies, Inc. (IDT, Leuven, Belgium) and are presented in Supplemental Table S2.

### In silico analyses

The prediction of coiled-coil motifs (Supplemental Fig. S1a, S1b) was performed using the DeepCoil online tool (Ludwiczak et al. 2019) (https://toolkit.tuebingen.mpg.de/tools/deepcoil).

The estimated protein mass was calculated as follows. Signal peptides for secretion were predicted using SignalP 5.0 software tool (Armenteros et al. 2019) (http://www.cbs.dtu.dk/services/SignalP/). Estimation of mature amino acid sequence was subsequently calculated using the ProtParam tool from the ExPASy web server (https://web.expasy.org/protparam/).

### SDS-PAGE and enzyme activity assays

Liquid culture filtrates of the control and mutant strains grown in media containing 1% citrus pectin, 1% D-xylose, or the combination of 1% citrus pectin and 0.075% (5 mM) D-xylose for 24 h were used to evaluate the produced extracellular CAZymes.

Twelve microliters of the culture filtrates were added to 4 μL loading buffer (10% of 1 M Tris–HCl, pH 6.8; 42% glycerol, 4% (w/v) SDS; 0.02% (w/v) bromophenol blue; 4% of 14.7 M mercaptoethanol), incubated at 85°C for 15 min, ice-cooled for 2 min and centrifuged at ~ 10,000 *× g* for 2 min. Finally, 15 μL of sample were loaded onto 12% (w/v) acrylamide SDS-PAGE gels calibrated with PageRuler prestained protein marker (Thermo Fisher Scientific, Waltham, MA, USA). Visualization was performed by silver staining (Chevallet et al. 2006), while documentation was done by using a HP Scanjet G2410 scanner. All samples were evaluated in biological duplicates.

Enzyme activities were performed by using the colorimetric *para*-nitrophenol (*p*NP) or azo-dye substrate assays in 96-well flat bottom microtiter plates. For *p*NP assays, 10 μL of supernatant samples were mixed with 10 μL of 0.1% 4-nitrophenyl β-D-glucopyranoside for β-glucosidase (BGL) activity or 4-nitrophenyl β-D-xylopyranoside for β-xylosidase (BXL) activity substrates, 50 μL of 50 mM NaAc (pH 5), and 30 μL of demineralized water in a final volume of 100 μL. Both *p*NP assays were measured after 1 h incubation at 30°C. The reactions were stopped by adding 100 μL of 0.25 M Na_2_CO_3_, and the absorption values were measured at 405 nm wavelength using a FLUOstar OPTIMA microplate reader (BMG Labtech, Ortenberg, Germany). For azo-dye substrate assays, 20 μL of supernatant samples were mixed with 30 μL of 100 mM NaAc (pH 4.6) and 50 μL of Azo-CM-Cellulose (Megazyme, Bray, Ireland) or Azo-Xylan (birchwood) (Megazyme, Bray, Ireland) substrate for endoglucanase (EGL) and endoxylanase (XLN) activity measurement, respectively. The reaction mixtures were incubated for 4 h at 30°C and were terminated by the addition of 250 μL of precipitation solution (4% NaAc*3H_2_O, 0.4% ZnAc, 76% EtOH, pH 5). The plates were centrifuged at 4°C, 1000 *× g* for 10 min. Supernatant samples were transferred to new microtiter plates, and absorption was measured at 600 nm wavelength using a FLUOstar OPTIMA microplate reader. All measurements were performed by using biological duplicates and technical triplicates.

### Saccharification test

Saccharification tests were performed in 96-well flat bottom microtiter plates. Each reaction had 50 mM sodium citrate (pH 5) containing 3% soybean hulls (SBH) or 3% CP mixed with 20 μL culture filtrate in a final volume of 250 μL. The reaction plates were incubated for 6 h at 30°C and 400 rpm. Reactions were stopped by heat inactivation for 15 min at 95°C. The reaction plates were centrifuged for 20 min at 3220 × *g*, and the supernatants were subsequently 10-fold diluted in MilliQ water prior to analysis. The experiment was performed using biological duplicates and technical triplicates.

Monosaccharides were analyzed from peak areas in HPAEC-PAD (Dionex ICS-5000 + system; Thermo Fisher Scientific, Waltham, MA, USA) equipped with CarboPac PA1 column (2×250 mm with 2×50 mm guard column; Thermo Fisher Scientific, Waltham, MA, USA). The column was pre-equilibrated with 18 mM NaOH followed by a multi-step gradient: 0–20 min, 18 mM NaOH; 20–30 min: 0–40 mM NaOH and 0–400 mM sodium acetate; 30–35 min, 40–100 mM NaOH and 400 mM to 1 M sodium acetate; and 35–40 min, 100 mM NaOH and 1 M to 0 M sodium acetate followed by re-equilibration of 18 mM NaOH for 10 min (20°C; flow rate: 0.30 mL min^−1^). Concentrations of 5–250 mM of D-xylose, D-galacturonic acid, and L-arabinose (Sigma-Aldrich, St. Louis, MO, USA) were used as standards for quantification. Blank samples containing 3% SBH or CP, with the addition of sterile MilliQ water instead of culture filtrates were measured as well. These values were subtracted from each corresponding saccharification sample result in order to exclude the amount of free sugar already present in the experimental condition.

### Proteomics sample preparation and analysis

Proteins from 600 μL cell-free liquid culture filtrates were precipitated by mixing them with two volumes of −20°C methanol, followed by overnight incubation at −20°C. The precipitated protein solution was centrifuged at 20800 × *g*, 4°C for 20 min. The supernatant was aspirated, and the pellet was washed once with 60% cold methanol in water and was resuspended in 6M urea, 100 mM ammonium bicarbonate pH 8 solution. An aliquot was subsequently taken for protein quantification performed colorimetrically using the RCDC kit assay (BioRad, Mississauga, Ontario). In total 7.5 μg of protein samples of biological duplicates were immobilized in acrylamide and processed for in-gel digestion with trypsin as previously described (Balliau et al. 2018). Dried digest peptide extracts were solubilized in a solution of 5% acetonitrile, 0.1% formic acid, and 4 fmol μL^−1^ of trypsin-digested bovine serum albumin (BSA) (Michrom, Auburn, CA) used as internal standard. Five microliters were analyzed by LC-MS/MS using an Easy-LC II Nano-HPLC system connected in-line with a Velos LTQ-Orbitrap mass spectrometer (Thermo Fisher Scientific, San Jose, CA). LC-MS/MS data peptide and protein identification were done using the *A. niger* NRRL3 protein sequence databases. Protein identification and quantification were performed using the Proteome Discoverer 2.4 (Thermo Fisher Scientific, Waltham, MA, USA) precursor ion quantitation workflow. Normalized individual protein area values were expressed as a fold value of the protein area value determined for the BSA internal standard. Data analysis was performed based on the percentage values of the total exoproteome.

### Statistical analysis

Statistical analyses were performed on all enzyme assays and saccharification experiments, which were carried out in biological duplicates and technical triplicates. Statistically significant differences (*p* value < 0.05) were determined using the one-way analysis of variance (ANOVA) and Tukey’s honestly significant difference (HSD) test (Supplemental Table S3). Analyses were done using STATGRAPHICS Centurion XVI Version 16.1.17 (www.statgraphics.com/centurion-xvi).

## Results

### The P*pgaX*-*hph* expression construct allows the screening of functional chimeric transcription factors

**Fig. 1:**
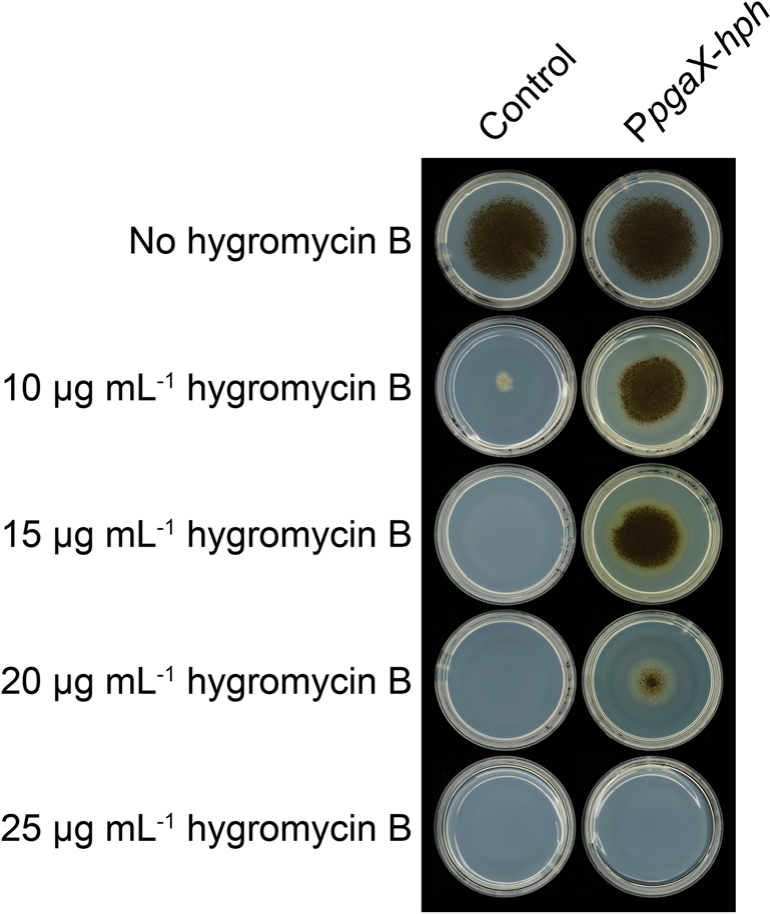
Hygromycin B resistance test of the *A. niger* P*pgaX*-*hph* reporter strain. The control (CBS 138852) and reporter CBS 147359 (CBS 138852 P*pgaX*-*hph*) strains were grown on media containing 25 mM D-galacturonic acid as sole carbon source in presence of increasing concentrations (10–25 μg mL^−1^) of antibiotic. Pictures were taken after 10 days of incubation at 30°C

The *pgaX* gene encoding an exopolygalacturonase has previously been shown to be under the control of GaaR in *A. niger* (Alazi et al. 2016; Kowalczyk et al. 2017b). Therefore, we selected the promoter of this gene as a target for screening the activity of GaaR-XlnR chimeric transcription factors, which are able to bind to a GaaR-specific DNA-binding site. The CRISPR/Cas9 system was used to delete the ORF of *pgaX*, and a repair template carrying the *hph* gene was used to replace the deleted *pgaX* gene. In order to ensure GaaR-mediated expression of *hph* under the control of *pgaX* promoter (P*pgaX*-*hph*), the reporter strain was grown on media containing D-galacturonic acid as sole carbon source supplemented with increasing concentrations of hygromycin B. Growth of the parental strain was severely impaired at 10 μg mL^−1^ of hygromycin B, whereas the reporter strain showed growth when the hygromycin B concentration was in the range of 10–20 μg mL^−1^ (Fig. 1). However, the reporter strain failed when higher concentrations of hygromycin B were applied. Based on these results, the concentration of 15 μg mL^−1^ hygromycin B was used for further screening purposes.

We constructed three different chimeric GaaR-XlnR models (168.1, 169.1, and 170.1) by fusion PCR to identify an efficient chimera. In all cases, the C-terminal region consisted of the amino acids 202-945 of XlnR, which includes the fungal transcription factor activation domain (Hasper et al. 2004). The three constructs differ in the GaaR N-terminal regions, which were selected based on prediction of putative coiled-coil elements (Supplemental Fig. S1a) and amino acid sequence conservation across a wide range of filamentous fungi (data not shown). In case of the chimeric model 168.1, the N-terminal 1-107 amino acid sequence of GaaR was fused together with the C-terminal region of XlnR (Supplemental Fig. S1c). The N-terminal sequence of GaaR retained its endogenous zinc-finger domain followed by a linker and a hypothetical coiled-coil sequence, as in silico predicted. For model 169.1, the N-terminal GaaR region consisted of the amino acids 1-193, carrying an additional putative coiled-coil region (Supplemental Fig. S1d), while in model 170.1, the GaaR fragment consisted of a larger N-terminal fragment of 1-229 amino acids (Supplemental Fig. S1e). The expression of each chimeric construct was driven by the endogenous *xlnR* promoter. The chimeric GaaR-XlnR mutants were generated in the P*pgaX*-*hph* background strain to assess the function of the chimeric constructs.

**Fig. 2:**
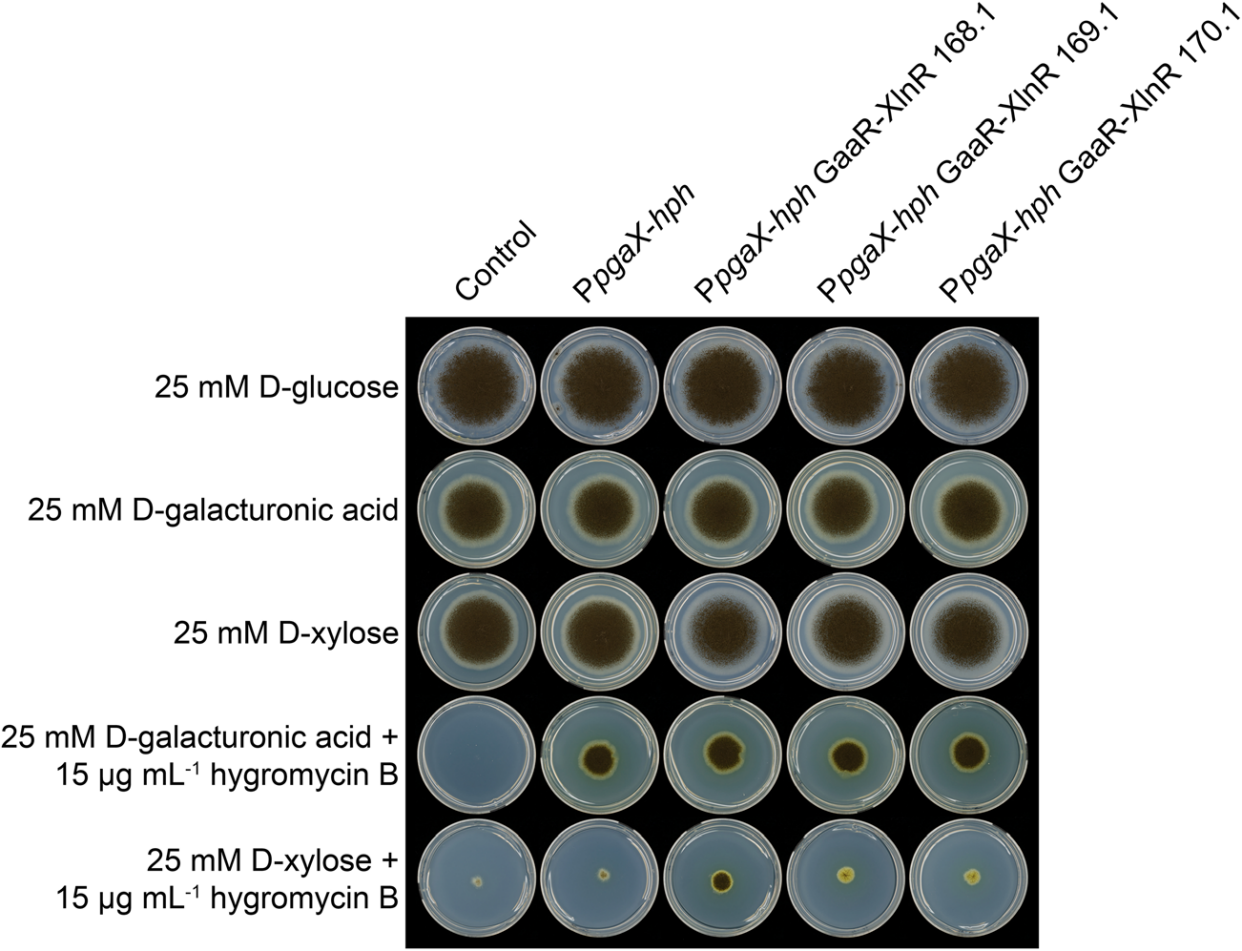
Functionality test of GaaR-XlnR chimeric transcription factor mutants. The growth of control (CBS 138852), P*pgaX*-*hph* reporter strain, and the P*pgaX*-*hph* strains carrying chimeric GaaR-XlnR constructs was tested on MM containing either 25 mM D-xylose or D-galacturonic acid as carbon source supplemented with 15 μg mL^−1^ hygromycin B. Plates without hygromycin B and with D-glucose as the sole carbon source were included as reference. Pictures were taken after 8 days of incubation at 30°C

Subsequently, the control (CBS 138852), P*pgaX*-*hph*, and mutants carrying each of the three chimeric GaaR-XlnR constructs in a P*pgaX*-*hph* background were tested for growth on media containing D-galacturonic acid or D-xylose supplemented with 15 μg mL^−1^ hygromycin B (Fig. 2). None of the strains showed reduced growth on the media containing 25 mM D-glucose, 25 mM D-galacturonic acid, or 25 mM D-xylose in the absence of hygromycin B. These results suggest that the tested strains do not display metabolic defects in the utilization of D-glucose, D-galacturonic acid, and D-xylose and that the differential growth observed on media supplied with 15 μg mL^−1^ hygromycin B is attributed to the presence of the drug. The P*pgaX*-*hph* reporter strain, as well as the chimeric mutants, showed substantial growth on D-galacturonic acid supplied with hygromycin B. When D-xylose was the sole carbon source, the chimeric mutants 169.1 and 170.1 showed growth comparable to that of the reporter strain after 8 days of incubation, suggesting insufficient expression of the reporter construct mediated through their chimeric GaaR-XlnR transcription factors. In contrast, the chimeric mutant 168.1 showed substantially improved growth and sporulation compared to the reporter strain and the other chimeric mutants. Thus, the chimeric model 168.1 was chosen for further characterization.

### The chimeric GaaR-XlnR mutant showed impaired XlnR activity

**Fig. 3:**
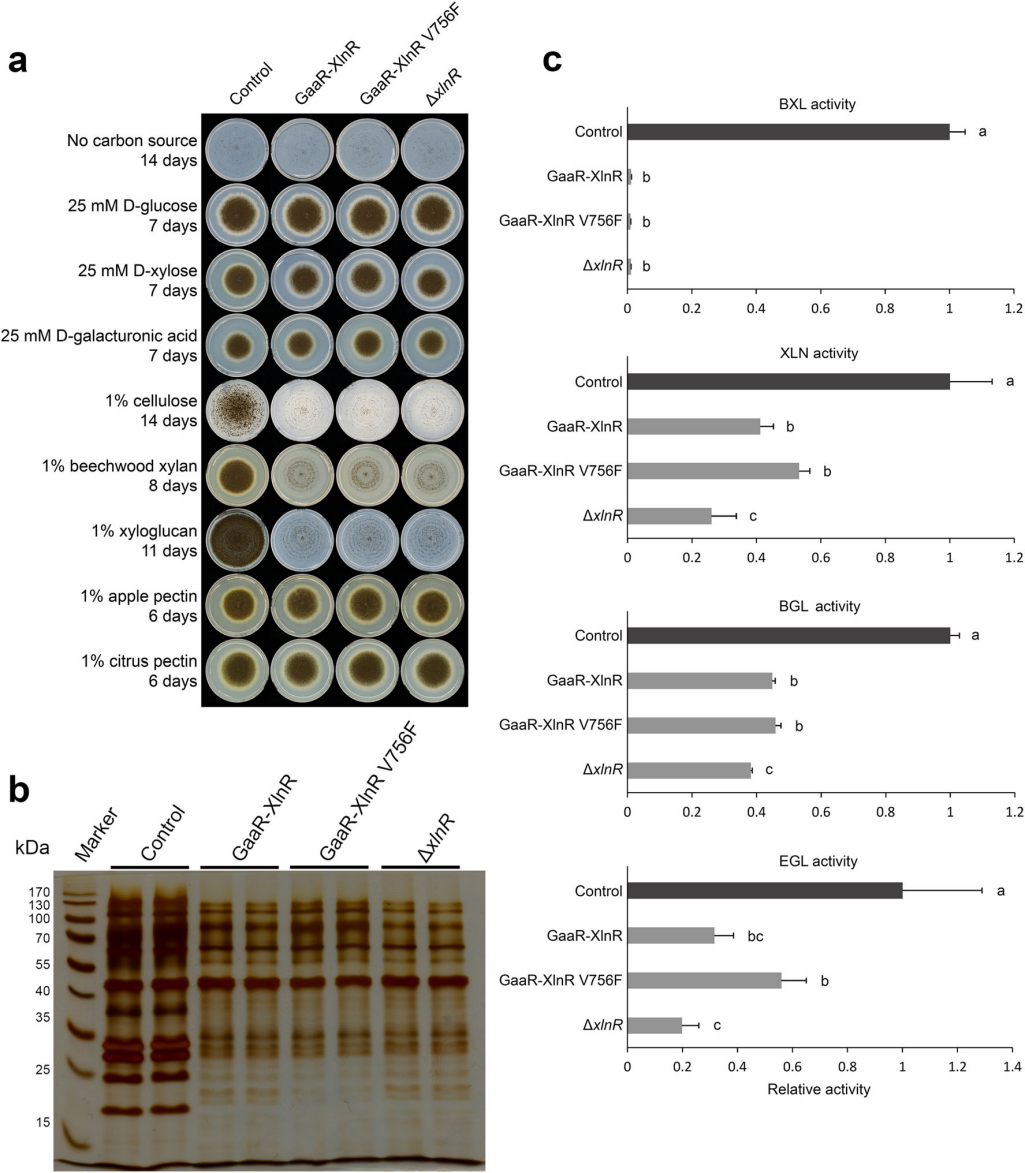
Phenotypic screening of GaaR-XlnR chimeric mutant strains. **a** Growth profile of *A. niger* control (CBS 138852), GaaR-XlnR, GaaR-XlnR V756F, and Δ*xlnR* strains on selected mono- and polysaccharides. All plates were incubated at 30°C for up to 14 days. Note that the Δ*xlnR* strain was included as a negative control for the loss of XlnR function. **b** Extracellular protein analysis of *A. niger* control (CBS 138852) and mutant strains. Supernatant filtrates were harvested from 2% wheat bran liquid cultures incubated at 30°C and 250 rpm for 24 h. **c** Enzyme activity assays of supernatant filtrates originated from 2% wheat bran liquid cultures after 24 h incubation at 30°C and 250 rpm. Graph bars represent normalized enzyme activity values, and letters (a–c) represent the statistical differences between samples within each specific enzyme assay. Samples showing different letters show significant differences among the strains, while samples sharing the same letters show no statistically significant differences (ANOVA and Tukey’s HDS test, *p* < 0.05)

Based on our initial screening, the GaaR-XlnR chimeric model 168.1 was used to generate a GaaR-XlnR chimeric mutant in the *A. niger* CBS 138852 background strain. Moreover, the CRISPR/Cas9 system was also used to generate a constitutively active form of the chimeric transcription factor by introducing a point mutation (V756F) in the C-terminal region of XlnR as described before (Kun et al. 2020). XlnR has been described to be regulated at the post-translational level through a proposed D-glucose inhibitory domain found in its C-terminal region, which is responsible for turning XlnR into an inactive state under repressing conditions (Hasper et al. 2004). The V756F mutation disturbs this inhibitory domain, keeping XlnR in a permanently active form (Hasper et al. 2004). Therefore, the constitutively active form of the chimeric transcription factor is independent of the presence of the activator (D-xylose), showing a clearer phenotype of the chimeric mutation. Growth profile results showed that the growth of the GaaR-XlnR and the GaaR-XlnR V756F mutants was comparable to that of the Δ*xlnR* mutant (Fig. 3a), indicating that the replacement of the XlnR N-terminal region for that of GaaR resulted in the loss of native XlnR function. The extracellular protein profile of these mutants grown on 2% wheat bran liquid cultures further supported this observation, since the production of the major endoxylanases found in the molecular mass range of 13–33 kDa (de Vries and Visser 2001) was highly reduced (Fig. 3b). Moreover, enzyme activity assays (Fig. 3c) confirmed the abolition of β-xylosidase (BXL) activity and the high reduction in endoxylanase activity (XLN) (Fig. 3c), which are both required for the efficient degradation of xylan and are (mainly) under the control of XlnR. The impaired growth on cellulose (Fig. 3a) also correlates with the overall reduction of cellulolytic activities, indicated by the β-glucosidase (BGL) and endoglucanase (EGL) activities (Fig. 3c).

### The GaaR-XlnR chimeric transcription factor recovers growth on pectin in a Δ*gaaR* strain

**Fig. 4:**
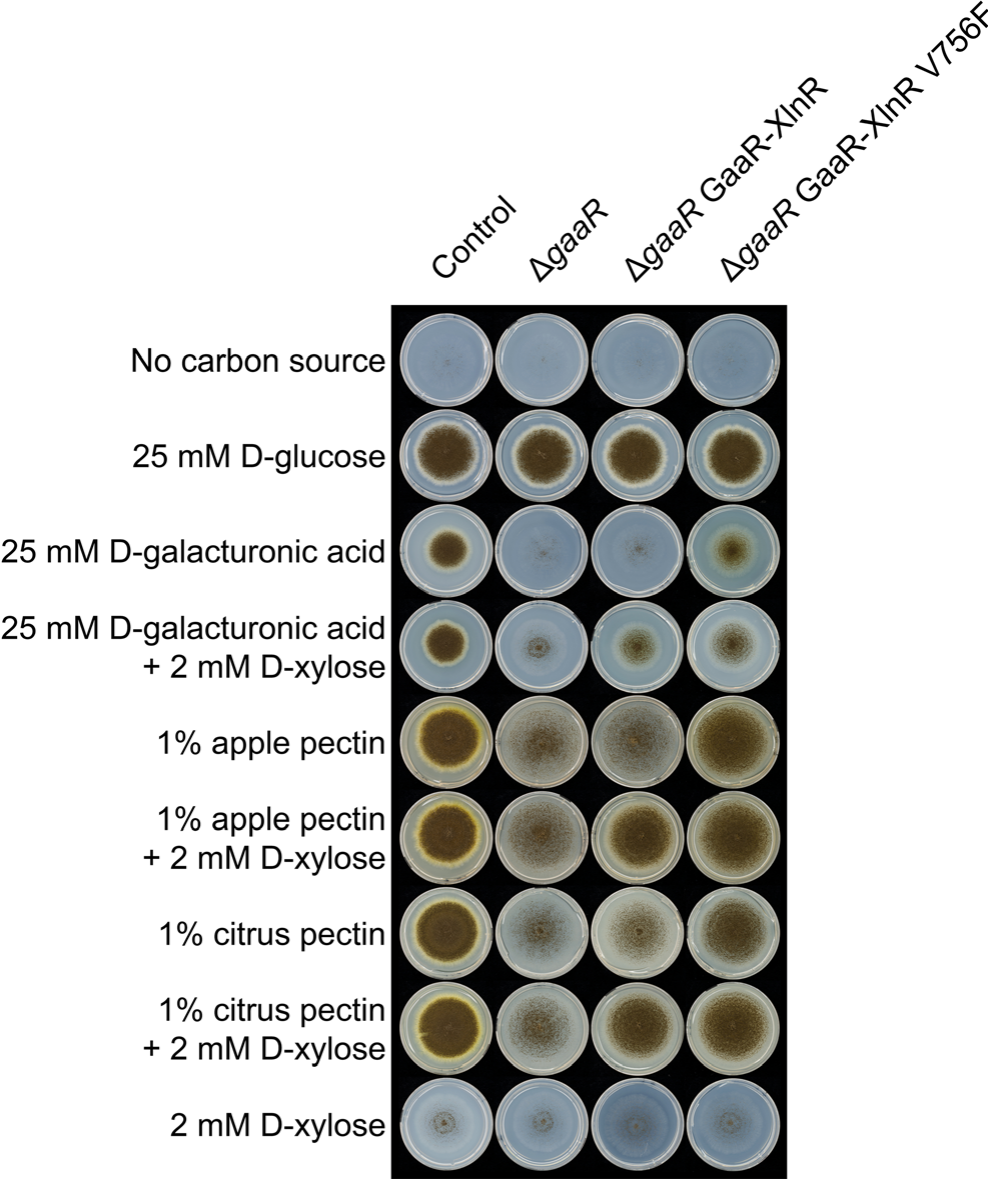
Growth test of GaaR-XlnR chimeric mutants on pectin and related substrates. The control (CBS 138852), Δ*gaaR*, Δ*gaaR* GaaR-XlnR, and Δ*gaaR* GaaR-XlnR V756F strains were grown on media containing 1% apple/citrus pectin and D-galacturonic acid. The test substrates were also supplemented with D-xylose for the induction of the chimeric transcription factor. All plates were incubated for 7 days at 30°C

To evaluate the pectinolytic activities mediated by a GaaR-XlnR chimeric transcription factor, the GaaR-XlnR and GaaR-XlnR V756F mutations were generated in a Δ*gaaR* background strain. Growth profiling (Fig. 4) showed abolished growth on D-galacturonic acid and highly reduced growth on apple and citrus pectin for Δ*gaaR*. The Δ*gaaR* GaaR-XlnR mutant showed comparable growth to Δ*gaaR* on 25 mM D-galacturonic acid, as well as on 1% apple pectin or 1% citrus pectin. However, the addition of 2 mM D-xylose resulted in substantial growth recovery on these substrates. In contrast, Δ*gaaR* GaaR-XlnR V756F improved growth compared to Δ*gaaR* even without the addition of 2 mM D-xylose, thus demonstrating the inducer-independent nature of this chimeric transcription factor. All strains showed minimal growth on 2 mM D-xylose as sole carbon source, indicating that the differential growth of chimeric mutants on substrates with or without 2 mM D-xylose is not attributed to the metabolism of D-xylose present in the media.

Despite the addition of D-xylose, none of the chimeric mutants showed fully recovered growth on 25 mM D-galacturonic acid.

### Chimeric GaaR-XlnR transcription factor activates the production of pectinolytic enzymes

**Fig. 5:**
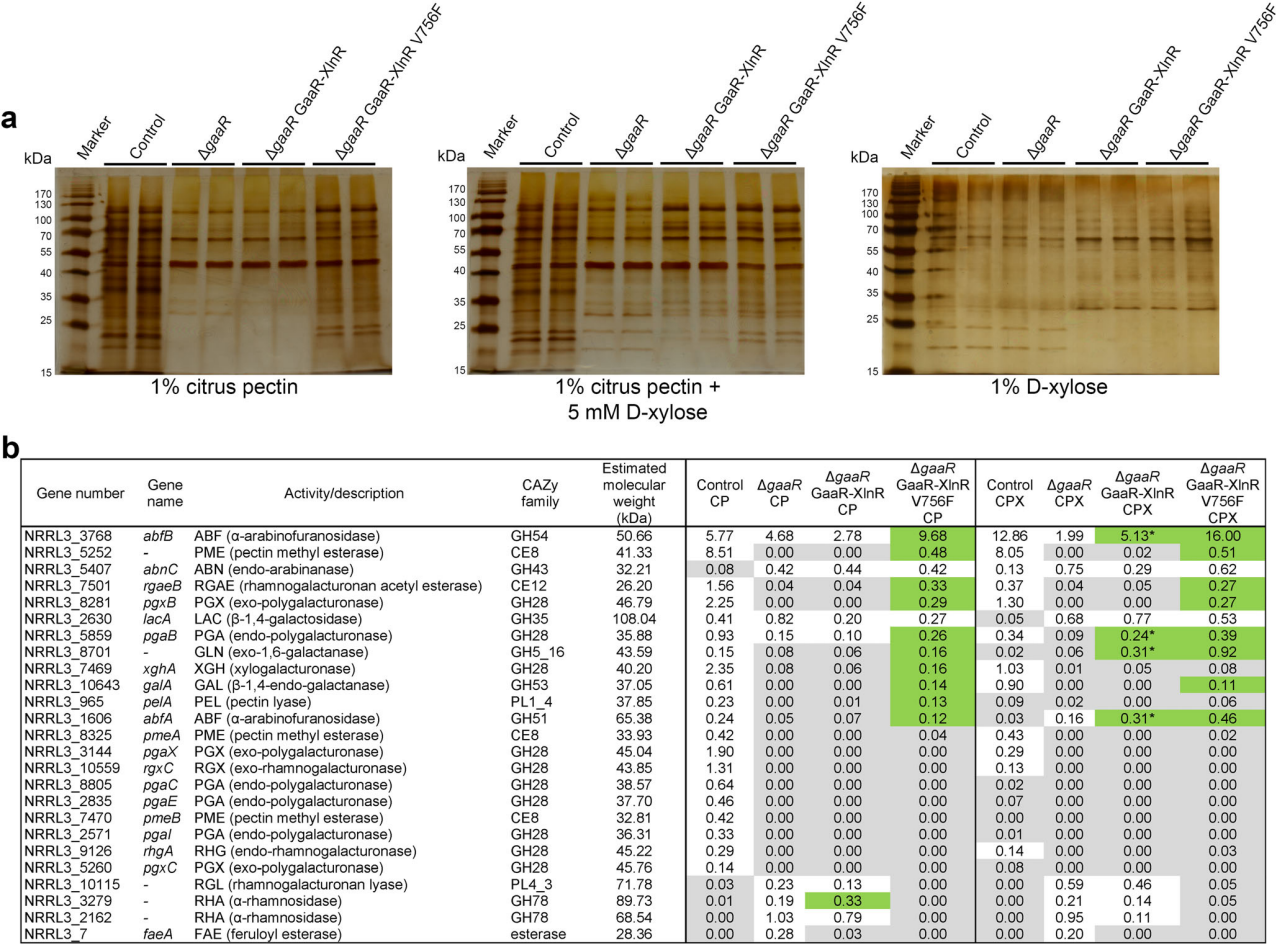
Exoproteome analysis of GaaR-XlnR chimeric mutants in Δ*gaaR* background strain. **a** SDS-PAGE analysis of supernatant samples. Supernatant filtrates of control (CBS 138852), Δ*gaaR*, and Δ*gaaR* GaaR-XlnR and Δ*gaaR* GaaR-XlnR V756F chimeric transcription factor mutants were harvested after 24 h incubation at 30°C and 250 rpm. Different liquid culture conditions are indicated in the figure. Samples are shown as biological duplicates. **b** Selected proteins associated with the degradation of pectin detected by proteomics analysis in the exoproteome of control (CBS 138852) and mutant strains. Samples were harvested from 1% citrus pectin (CP) or 1% citrus pectin + 5 mM D-xylose (CPX) liquid cultures. Protein abundance is represented as percentage of the total exoproteome. Proteins with an abundance < 0.1% of the total exoproteome are indicated in gray cells. Proteins showing > 1.5-fold increase in the chimeric mutants compared to the Δ*gaaR* strain are indicated in green cells. The asterisk (*) indicates the proteins which showed increased abundance compared to the Δ*gaaR* strain only when the media was supplemented with D-xylose. The total exoproteome data are found in Supplementary Data 1

To evaluate which pectinolytic proteins are produced by the chimeric GaaR-XlnR mutants, the *gaaR* deficient mutants carrying the chimeric transcription factor were cultivated in 1% citrus pectin and 1% citrus pectin + 5 mM D-xylose liquid media. SDS-PAGE analysis showed comparable protein production for Δ*gaaR* and Δ*gaaR* GaaR-XlnR after 24h incubation in 1% citrus pectin (Fig. 5a). In contrast, Δ*gaaR* GaaR-XlnR V756F showed substantially improved protein production compared to both Δ*gaaR* and Δ*gaaR* GaaR-XlnR. However, the protein pattern of the mutant carrying the constitutively active chimeric transcription factor did not show complete recovery of extracellular enzymes compared to the control (CBS 138852) strain. When cultivated on 1% citrus pectin + 5 mM D-xylose or on 1% D-xylose, both mutants carrying the chimeric GaaR-XlnR construct showed a comparable extracellular protein profile (Fig. 5a). This result correlates with the observed growth phenotype (Fig. 4), indicating the induction of the chimeric GaaR-XlnR in the presence of D-xylose and the inducer-independent activity of the constitutively active GaaR-XlnR V756F form.

Proteomic analysis showed partial or full recovery of the production of several proteins involved in pectin degradation in Δ*gaaR* GaaR-XlnR V756F compared to Δ*gaaR* (Fig. 5b) when cultured on citrus pectin (CP) and citrus pectin supplemented with D-xylose (CPX). Several proteins (e.g., NRRL3_5252, RgaeB, PgxB, and GalA) increased in levels in both conditions (CP and CPX) in the constitutively active mutant. However, the addition of D-xylose to the culture media did not result in increased production of these proteins in Δ*gaaR* GaaR-XlnR. In contrast, an endo-polygalacturonase (PgaB), an endo-1,6-β-galactanase (NRRL3_8701), and two α-L-arabinofuranosidases (AbfA and AbfB) showed substantially increased abundance in both CP and CPX filtrates of Δ*gaaR* GaaR-XlnR V756F, as well as in the CPX filtrate of Δ*gaaR* GaaR-XlnR. The arabinofuranosidase AbfB showed the highest abundance in the exoproteome of both Δ*gaaR* GaaR-XlnR and Δ*gaaR* GaaR-XlnR V756F filtrates. Interestingly, both the cellobiohydrolase CbhB and the glucoamylase GlaA showed upregulation in the CPX filtrate exoproteome of both chimeric mutants compared to Δ*gaaR* (Supplementary Data 1). These proteins were also the most abundant ones in the exoproteome next to AbfB.

### The GaaR-XlnR V756F chimeric transcription factor improves the release of D-galacturonic acid and L-arabinose from pectin

**Fig. 6:**
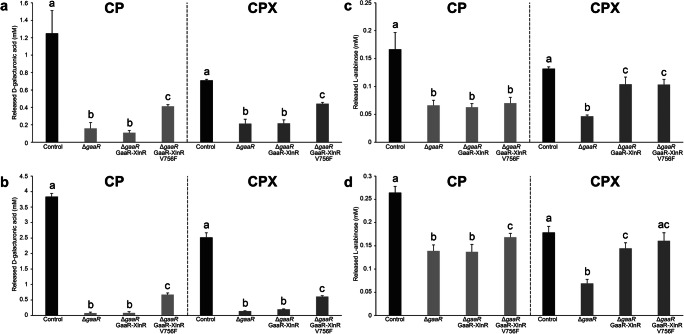
Saccharification analysis of 3% soybean hulls and 3% citrus pectin substrates by *A. niger* control (CBS 138852) and mutant strain supernatant filtrates. Supernatant filtrates were harvested from 1% citrus pectin (CP) or 1% citrus pectin + 5 mM D-xylose (CPX) liquid cultures. Graphs illustrate the amount of released D-galacturonic acid from 3% soybean hulls (**a**) and 3% citrus pectin (**b**), as well as the released L-arabinose from 3% soybean hulls (**c**) and 3% citrus pectin (**d**). Letters (a–c) represent the statistical differences between samples within each specific saccharification assay. Samples showing different letters show significant differences among the strains, while samples sharing the same letters show no statistically significant differences (ANOVA and Tukey’s HDS test, *p* < 0.05)

The saccharification and sugar release analysis of 3% soybean hulls (SBH) and 3% CP were performed to evaluate the enzymatic activities present in the CP and CPX liquid culture filtrates of the control (CBS 138852) and mutant strains after 24 h incubation. Soybean hulls has been selected as a crude substrate for saccharification due to its high pectin content represented by the abundant presence of D-galactose, L-arabinose, and D-galacturonic acid in its composition (Mäkelä et al. 2017). The amount of released D-xylose from SBH was low (< 0.1 mM) for all strains, including the control (Supplemental Fig. S2). However, no D-xylose has been detected when either the filtrate of Δ*gaaR* GaaR-XlnR or Δ*gaaR* GaaR-XlnR V756F was used. None of the filtrates resulted in the release of D-xylose from 3% CP, most likely due to the low abundance of this sugar in its composition. Surprisingly, the release of D-galacturonic acid from either 3% SBH (Fig. 6a) or 3% CP (Fig. 6b) did not improve in case of Δ*gaaR* GaaR-XlnR compared to Δ*gaaR* at any of the conditions tested. In contrast, Δ*gaaR* GaaR-XlnR V756F showed consistently improved D-galacturonic acid release when either the CP or CPX filtrates were used (Fig. 6a, b). However, this amount was lower than that of the control strain. On the other hand, the release of L-arabinose from 3% SBH (Fig. 6c) and 3% CP (Fig. 6d) was comparable for all mutant strains when the CP filtrates were used, with a slight improvement of release in case of the constitutive GaaR-XlnR V756F from 3% CP (Fig. 6d). The chimeric mutants showed the most significant improvement of L-arabinose release compared to Δ*gaaR* when the CPX filtrates were used.

## Discussion

In this study, we tested three versions of GaaR-XlnR chimeric transcription factor mutants to generate an *A. niger* strain that is able to produce pectinolytic enzymes by using D-xylose as inducing sugar.

The majority of fungal transcriptional activators belong to the Zn_2_Cys_6_ cluster family proteins. These proteins usually carry the DNA-binding motif at the N-terminal region, while the activation domain is found in the C-terminal region of the protein (Pfeifer et al. 1989). It has been reported that the binding specificity of a Zn_2_Cys_6_ transcription factor is affected by the linker region on the C-terminal end of the zinc-finger motif (Corton 1989; Johnston and Dover 1987). However, it has also been shown that binding specificity can be influenced by the N-terminal end of a putative dimerization element represented by a coiled-coil following the linker region (Marmorstein and Harrison 1994). Considering these observations, we decided to fuse the C-terminal region of XlnR (202-945 aa) together with three different N-terminal GaaR fragments, all of them including the zinc-finger motif (26-53 aa), linker region (54-64 aa), and the neighboring putative coiled-coil element (65-85 aa) (Supplemental Fig. S1).

To simplify the screening of chimeric GaaR-XlnR constructs, we constructed a P*pgaX*-*hph* reporter strain. The utilization of the endogenous *pgaX* promoter-reporter system for the analysis of GaaR-mediated activation of a selection marker gene (*amdS*) has previously been reported (Niu et al. 2015). In our study, we used the hygromycin B phosphotransferase gene (*hph*) for screening purposes. It has been reported that *A. niger* is able to grow when the concentration of hygromycin B is even higher than 100 μg mL^−1^ (Punt et al. 1987). However, in most cases, the *hph* gene is expressed under the control of a strong constitutive promoter, such as P*gpdA* from *Aspergillus nidulans*. Initial screening indicated that the GaaR (1-107 aa)-XlnR (202-945 aa) chimera (model 168.1) showed the best resistance to hygromycin B when induced by D-xylose (Fig. 2), indicating that the presence of additional GaaR sequence elements (models 169.1, 170.1) might result in the loss of function and/or reduced stability of the chimeric transcription factor. However, the slow growth of this mutant indicates that the chimeric transcription factor only partially activates the expression of the reporter gene compared to the endogenous GaaR. This result correlates with the observation that certain chimeric constructs show relatively low affinity towards some specific target genes (Witte and Dickson 1990).

Phenotypic analysis indicated the high reduction or even abolition of enzymatic activities affected by XlnR in the GaaR-XlnR and GaaR-XlnR V756F mutants (Fig. 3). Although the XLN, BGL, and EGL enzyme activities were highly reduced, they were not completely abolished in any of the chimeric mutants, most likely due to the involvement of other (hemi-)cellulolytic transcription factors, such as ClrA and/or ClrB in the regulation of these activities in *A. niger* (Raulo et al. 2016). However, the residual cellulolytic activities were not sufficient to support growth on cellulose (Fig. 3a). Interestingly, the XLN, BGL, and EGL activities were in general slightly higher than in the Δ*xlnR* strain, which might indicate residual direct or indirect activation of the corresponding genes. The growth on pectin was not increased in the chimeric mutant strains compared to the control, suggesting that the chimeric transcription factor does not improve the expression of pectinolytic genes in the presence of an active GaaR. However, the activation of the pectinolytic system by the chimeric transcription factor was more prominent when the chimeric mutation was introduced in a Δ*gaaR* background strain. The addition of 2 mM D-xylose to the growth test substrates clearly shows the activation of the GaaR-XlnR chimeric transcription factor, which subsequently resulted in a substantial growth recovery on pectin. The chimeric mutant was able to grow on D-galacturonic acid after induction with D-xylose, but growth was only partially recovered. This indicates that the chimeric transcription factor is also able to activate all the essential genes encoding for the D-galacturonic acid catabolism. However, the artificial transcription factor might not have sufficient expression level or might not be stable enough to maintain a metabolic flux similar to the wild type. One alternative to improve the overall activity of the chimeric transcription factor would be the utilization of a strong constitutive promoter, as done in previous studies for the expression of other artificial transcription factors (Gao et al. 2017; Yamashita et al. 2021; Zhang et al. 2016, 2017), resulting in higher enzymatic activities.

The exoproteome patterns (Fig. 5a) of Δ*gaaR* GaaR-XlnR and Δ*gaaR* GaaR-XlnR V756F correlate with the growth profile results (Fig. 4), indicating that the constitutively active version of the chimeric transcription factor can activate its target genes in the absence of the inducing sugar. Proteomics analysis confirmed the activation of pectinolytic genes by the GaaR-XlnR chimeric transcription factor, showing substantially increased abundance of pectinolytic enzymes in the constitutively active Δ*gaaR* GaaR-XlnR V756F mutant compared to Δ*gaaR*. Of the proteins that showed increased abundance by Δ*gaaR* GaaR-XlnR in the CPX condition, AbfA (Alazi et al. 2016; Kowalczyk et al. 2017b) and PgaB (Kowalczyk et al. 2017b) have previously been described to be controlled by GaaR. The low abundance of AbfB in Δ*gaaR* compared to the control and the substantially increased abundance in the chimeric transcription factor mutants in the CPX condition indicates that production of this enzyme is also dependent on GaaR-mediated induction of the corresponding gene. Although GaaR is responsible for the production of most enzymes involved in the degradation of the polygalacturonic acid backbone (Kowalczyk et al. 2017b), in our experimental conditions, especially in the CPX medium, the expression of arabinanolytic genes was the most prominent. The molecular weight of the pectinolytic proteins found in the supernatants of the chimeric mutants have been estimated in this study (Fig. 5b), but due to post-translational modifications such as glycosylation, the estimated molecular weight of the proteins detected by proteomics analysis and the exoproteome pattern observed by SDS-PAGE analysis cannot be directly correlated. However, the molecular weight of the α-L-arabinofuranosidases AbfA and AbfB have been experimentally determined to be 83 kDa and 67 kDa, respectively (van der Veen et al. 1991), which appear at a higher intensity on the SDS-PAGE profile of the chimeric mutants when D-xylose is present in the liquid media (Fig. 5a).

The saccharification results also support the activation of arabinofuranosidases in the chimeric mutants. Interestingly, both CbhB and GlaA showed significant increase in abundance in the chimeric mutants, which is most likely an indirect effect of the chimeric mutation.

In conclusion, in this study, we report the utilization of CRISPR/Cas9 genome editing to generate a GaaR-XlnR chimeric transcription factor by precise on-site mutagenesis in *A. niger* for the first time. This artificial transcription factor was able to recover lost GaaR functions when induced by D-xylose. Moreover, the alteration of the specificity of the endogenous XlnR resulted in the downregulation of several (hemi-)cellulolytic enzymes due to the loss of XlnR activity, which can reduce the purification costs of pectinase-rich enzyme cocktails. Even though the chimeric mutant showed the upregulation of several pectinolytic proteins compared to the Δ*gaaR* strain, the abundance was in general lower than that of the control strain. These results may indicate that the expression level or the stability of the chimeric transcription factor could be improved. The utilization of a strong constitutive promoter for the expression of GaaR-XlnR might further improve the expression level of this artificial transcription factor and could possibly result in enriched pectinolytic enzyme cocktails when grown on D-xylose or xylan-rich substrates, with all the benefits that this would entail at the biotechnological and industrial level.

## Supplementary Information

ESM 1 (XLSX 127 kb)

ESM 2 (PDF 5706 kb)

## Data Availability

The mass spectrometry proteomics data have been deposited to the ProteomeXchange Consortium via the PRIDE (Perez-Riverol et al. 2019) partner repository with the dataset identifier PXD025383 and 10.6019/PXD025383 (http://www.ebi.ac.uk/pride/archive/projects/PXD025383).

## Declarations

This article does not contain any studies with human participants or animals performed by any of the authors.
