# Supplementary material for: The chimeric GaaR-XlnR transcription factor induces pectinolytic activities in the presence of D-xylose in Aspergillus niger
Source: Appl Microbiol Biotechnol. 2021 Jul 8;105(13):5553–64. doi: 10.1007/s00253-021-11428-2 (PMC8285313; doi:10.1007/s00253-021-11428-2)
Supplement: Supplementary file 2 — (PDF 5706 kb) [file 253_2021_11428_MOESM2_ESM.pdf]

Supplementary Material

Journal name:
Applied Microbiology and Biotechnology

Title:
The chimeric GaaR-XlnR transcription factor induces pectinolytic activities in the presence of D-xylose in *Aspergillus niger*

Authors:
Roland S. Kun[1], Sandra Garrigues[1], Marcos Di Falco[2], Adrian Tsang[2], Ronald P. de Vries[1]*

Affiliation:
[1]Fungal Physiology, Westerdijk Fungal Biodiversity Institute & Fungal Molecular Physiology, Utrecht University, Uppsalalaan 8, 3584 CT Utrecht, The Netherlands
[2]Centre for Structural and Functional Genomics, Concordia University, 7141 Sherbrooke Street West, Montreal, Quebec H4B 1R6, Canada.

***Corresponding author:**
Ronald P. de Vries
e-mail: r.devries@wi.knaw.nl

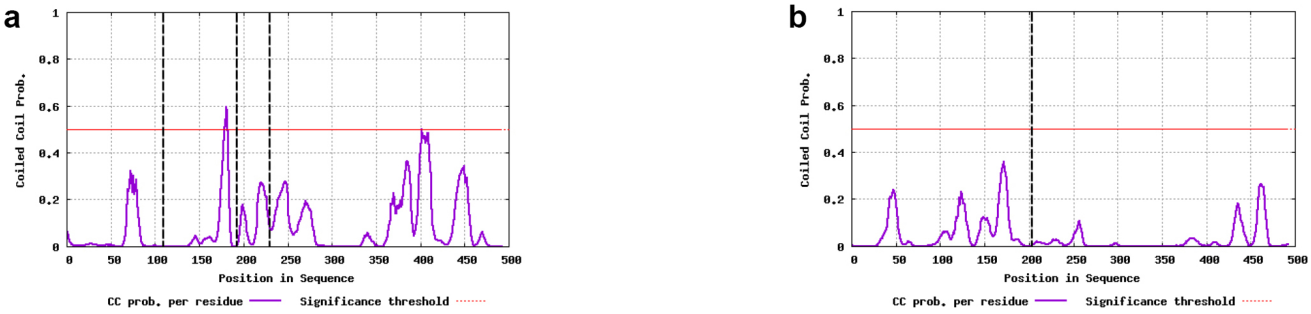

c

MSRPQVSIDRLTPRRVHAEPRESMNCKSCRKRKIKCNRLRPSCEACKVFQCPC**IYDAVPKKRGP**KTDVLEALLKRVDGLEKRLQEDSPISPT
SATSPVKSMDQQLLPGQDVNGTYDSAFESHHLSSQPSHMQHASTAGISGLHESQTAPSHSQSSLGTTIDAMHLNHFNTMNDSGRPAMSIS
DLRSLPPSVLPPQGLSSGYNASAFALVNPQEPGSPANQFRLGSSAENPTAPFLGLSPPGQSPGWLPLPSPSPANFPSFSLHPFSSTLRYPVLQPVLPHI
ASIIPQSLACDLLDVYFTSSSSSHLSPLSPYVVGYIFRKQSFLHPTKPRICSPGLLASMLWVAAQTSEAAFLTSPPSARGRVCQKLLELTIGLLRPLVHG
PATGEASPNYAANMVINGVALGGFGVSMDQLGAQSSATGAVDDVATYVHLATVVSASEYKAASMRWWTAAWSLARELKLGRELPPNVSHARQDG
ERDGDGEADKRHPPTLITSLGHGSGSSGINVTEEEREERRRLWWLLYATDRHLALCYNRPLTLLDKECGGLLQPMNDDLWQVGDFAAAAYRQVGP
PVECTGHSMYGYFLPLMTILGGIVDLHHAENHPRFGLAFRNSPEWERQVLDVTRQLDTYGRSLKEFEARYTSNLTLGATDNEPVVEGAHLDHT
SPSGRSSSTVGSRVSESIVHTRMVVAYGTHIMHVLHILLAGKWDPVNLLEDHDLWISSESFVSAMSHAVGAAEAAAEILEYDPDLSFMPFFFGI
YLLQGSFLLLLAADKLQGDASPSVVRACETIVRAHEACVVTLNTEYQRTFRKVMRSALAQVRGRIPEDFGEQQQRRREVLALYRWSGDGSGLAL

d

MSRPQVSIDRLTPRRVHAEPRESMNCKSCRKRKIKCNRLRPSCEACKVFQCPC**IYDAVPKKRGP**KTDVLEALLKRVDGLEKRLQEDSPISPTSATSPVKSMDQQLL
PSASTPFQFPTAPHGFRPPPQSQPPPQQHRLPDAMLDTYFTRLHGKPYWILDETATRQRHQHGQLPMHLSMAIYALTLRYTTPNPPQGQDVNGTYDSAFESH
HLSSQPSHMQHASTAGISGLHESQTAPSHSQSSLGTTIDAMHLNHFNTMNDSGRPAMSISDLRSLPPSVLPPQGLSSGYNASAFALVNPQEPGSPANQFRLGS
SAENPTAPFLGLSPPGQSPGWLPLPSPSPANFPSFSLHPFSSTLRYPVLQPVLPHIASIIPQSLACDLLDVYFTSSSSSHLSPLSPYVVGYIFRKQSFLHPTKPRIC
SPGLLASMLWVAAQTSEAAFLTSPPSARGRVCQKLLELTIGLLRPLVHGPATGEASPNYAANMVINGVALGGFGVSMDQLGAQSSATGAVDDVATYVHLATV
VSASEYKAASMRWWTAAWSLARELKLGRELPPNVSHARQDGERDGDGEADKRHPPTLITSLGHGSGSSGINVTEEEREERRRLWWLLYATDRHLALCYNRPLT
LLDKECGGLLQPMNDDLWQVGDFAAAAYRQVGPPVECTGHSMYGYFLPLMTILGGIVDLHHAENHPRFGLAFRNSPEWERQVLDVTRQLDTYGRSLKEFEAR
YTSNLTLGATDNEPVVEGAHLDHTSPSGRSSSTVGSRVSESIVHTRMVVAYGTHIMHVLHILLAGKWDPVNLLEDHDLWISSESFVSAMSHAVGAAEAAAEILEYD
PDLSFMPFFFGIYLLQGSFLLLLAADKLQGDASPSVVRACETIVRAHEACVVTLNTEYQRTFRKVMRSALAQVRGRIPEDFGEQQQRRREVLALYRWSGDGSGLAL

e

MSRPQVSIDRLTPRRVHAEPRESMNCKSCRKRKIKCNRLRPSCEACKVFQCPC**IYDAVPKKRGP**KTDVLEALLKRVDGLEKRLQEDSPISPTSATSPVKSM
DQQLLPSASTPFQFPTAPHGFRPPPQSQPPPQQHRLPDAMLDTYFTRLHGKPYWILDETATRQRHQHGQLPMHLSMAIYALTLRYTTPNPPQGSLE
YARQARRLVDIDNPSIEGTQSLLLLCHTFFAYGQDVNGTYDSAFESHHLSSQPSHMQHASTAGISGLHESQTAPSHSQSSLGTTIDAMHLNHFNTMND
SGRPAMSISDLRSLPPSVLPPQGLSSGYNASAFALVNPQEPGSPANQFRLGSSAENPTAPFLGLSPPGQSPGWLPLPSPSPANFPSFSLHPFSSTL
RYPVLQPVLPHIASIIPQSLACDLLDVYFTSSSSSHLSPLSPYVVGYIFRKQSFLHPTKPRICSPGLLASMLWVAAQTSEAAFLTSPPSARGRVCQKL
LELTIGLLRPLVHGPATGEASPNYAANMVINGVALGGFGVSMDQLGAQSSATGAVDDVATYVHLATVVSASEYKAASMRWWTAAWSLARELKLGRELP
PNVSHARQDGERDGDGEADKRHPPTLITSLGHGSGSSGINVTEEEREERRRLWWLLYATDRHLALCYNRPLTLLDKECGGLLQPMNDDLWQVGD
FAAAAYRQVGPPVECTGHSMYGYFLPLMTILGGIVDLHHAENHPRFGLAFRNSPEWERQVLDVTRQLDTYGRSLKEFEARYTSNLTLGATDNEPV
VEGAHLDHTSPSGRSSSTVGSRVSESIVHTRMVVAYGTHIMHVLHILLAGKWDPVNLLEDHDLWISSESFVSAMSHAVGAAEAAAEILEYDPDLSFMPFFF
GIYLLQGSFLLLLAADKLQGDASPSVVRACETIVRAHEACVVTLNTEYQRTFRKVMRSALAQVRGRIPEDFGEQQQRRREVLALYRWSGDGSGLAL

Figure S1 Sequence analysis of *A. niger* GaaR and XlnR transcription factors. **a**, **b** Prediction of putative coiled-coil regions in GaaR (**a**) and XlnR (**b**) amino acid sequence. Vertical black lines indicate the approximate position of the terminal amino acid of the N-terminal region of GaaR (**a**) and the starting amino acid of the C-terminal region of XlnR (**b**). **c-e** Amino acid sequence of chimeric GaaR-XlnR transcription factor model 168.1 (**c**), 169.1 (**d**) and 170.1 (**e**). The zinc-finger domain is indicated in green letters and the linker region in bold letters. The predicted coiled coil (Ludwiczak et al. 2019), which is putatively responsible for dimerization is indicated with underlined letters. The 202-945 aa C-terminal region of XlnR is indicated in red.

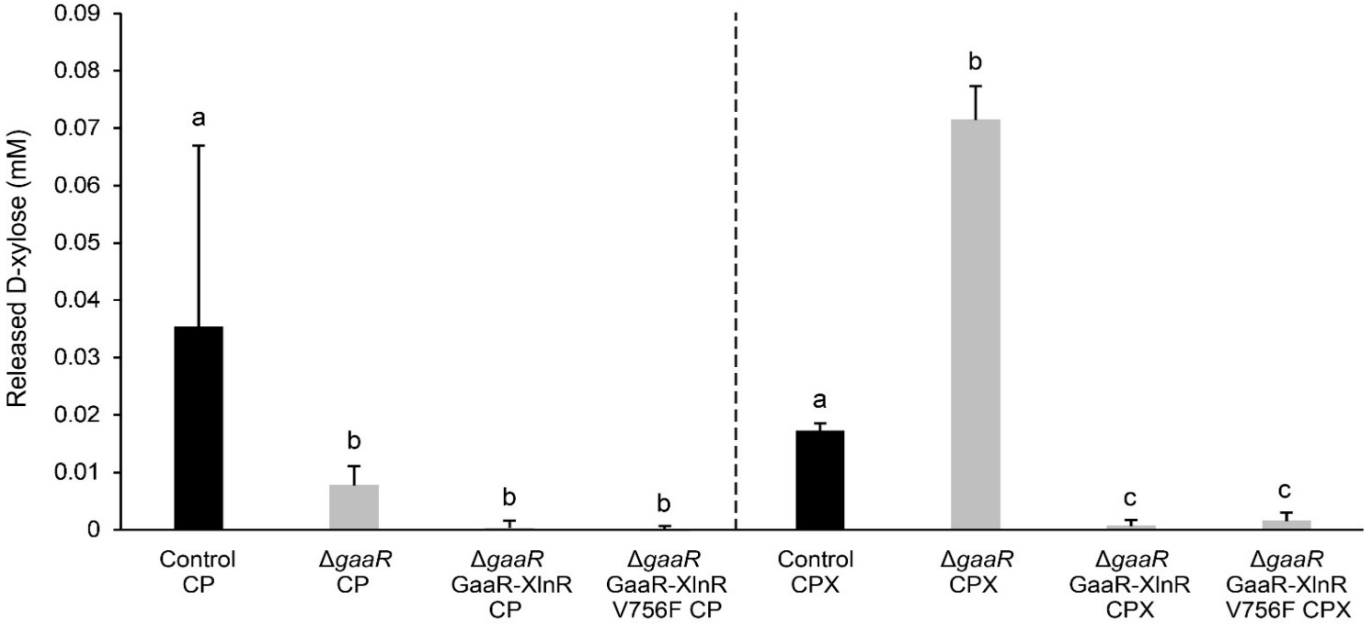

Figure S2 D-xylose released from 3% soybean hulls by *A. niger* control (CBS 138852) and mutant strains. Supernatant filtrates of tested strains originated from 1% citrus pectin (CP) or 1% citrus pectin + 5 mM D-xylose (CPX) liquid cultures.

Table S1 *Aspergillus niger* strains used in this study.

Strain ID	Genotype	CBS number	Reference
Control	*cspA1 kusA*::*amdS pyrG*$^{-}$	CBS 138852	Meyer et al. 2007
P*pgaX*-*hph*	CBS 138852 P*pgaX*-*hph*	CBS 147359	This study
P*pgaX*-*hph* GaaR-XlnR 168.1	CBS 138852 P*pgaX*-*hph* x*lnR*::*gaaR*-*xlnR* 168.1	CBS 147360	This study
P*pgaX*-*hph* GaaR-XlnR 169.1	CBS 138852 P*pgaX*-*hph* *xlnR*::*gaaR*-*xlnR* 169.1	CBS 147361	This study
P*pgaX*-*hph* GaaR-XlnR 170.1	CBS 138852 P*pgaX*-*hph* *xlnR*::*gaaR*-*xlnR* 170.1	CBS 147362	This study
GaaR-XlnR	CBS 138852 *xlnR*::*gaaR*-*xlnR*	CBS 147363	This study
GaaR-XlnR V756F	CBS 138852 *xlnR*::*gaaR*-*xlnR* V756F	CBS 147364	This study
Δ*gaaR* GaaR-XlnR	CBS 138852 Δ*gaaR* *xlnR*::*gaaR*-*xlnR*	CBS 147365	This study
Δ*gaaR* GaaR-XlnR V756F	CBS 138852 Δ*gaaR* *xlnR*::*gaaR*-*xlnR* V756F	CBS 147366	This study
Δ*xlnR*	CBS 138852 Δ*xlnR*	CBS 145447	Kun et al., unpublished
Δ*gaaR*	CBS 138852 Δ*gaaR*	CBS 146901	Garrigues et al., unpublished

Table S2 Primers used in this study. Homology flanks are highlighted in red.

Primer name	Sequence	Description
**Primers for construction of repair templates**		
xlnR N-term-5F	TGCCTGCTGTTTGCTTCTCG	for amplification of 5’ flank region of *xlnR* N-terminal region
xlnR N-term-5R	ACGGTCGATGCTGACCTGGGGGCGGGACATCCTCACCGGGCGGAACAATC	
xlnR N-term-3F 1	GTCAAATCCATGGACCAGCAACTGCTGCCAGGACAAGACGTGAACGGCAC	for amplification of 3’ flank region of *xlnR* N-terminal region model 1
xlnR N-term-3R	AGTTGCTACATCATCCACGGC	
xlnR N-term-3F 2	TGTAGATATACGACGCCGAATCCACCGCAAGGACAAGACGTGAACGGCAC	for amplification of 3’ flank region of *xlnR* N-terminal region model 2
xlnR N-term-3R	AGTTGCTACATCATCCACGGC	
xlnR N-term-3F 3	CTATTGCTCTGCCATACCTTTTTCGCCTATGGACAAGACGTGAACGGCAC	for amplification of 3’ flank region of *xlnR* N-terminal region model 3
xlnR N-term-3R	AGTTGCTACATCATCCACGGC	
gaaR N-term-F	TCGCACTGTCTAACCACATCC	for amplification of *gaaR* N-terminal region
gaaR N-term-R	GATACAAGTCCAACGCCAGG	
xlnR DBD-NEST-F	GAAAATAGTCCACCGTGTCTGG	for 5’ + 3’ flank fusion of *pgaX* repair template
xlnR DBD-NEST-R	ACGCCATTGATGACCATATTCG	
pgaX -5F	TTGCTTGCCAGTGTTGTAGACC	for amplification of 5’ flank region of *pgaX*
pgaX-5R	CTCGACAGACGTCGCGGTGAGTTCAGGCATTGTGAGAGATTGACAGGTGACAAG	
pgaX-3F	CCCAGCACTCGTCCGAGGGCAAAGGAATAGATCAAGATGCCTGTTATTATGCG	for amplification of 3’ flank region of *pgaX*
pgaX-3R	CAGTACAACAAAAATCAAGCCATCC	
hph- F	CACCATGCCTGAACTCACC	for amplification of *hph*
hph- R	CTATTCCTTTGCCCTCGGACG	
pgaX -NEST-F	GAAACCATTGAGATCGGCTTATGTG	for 5’ + 3’ flank fusion of *pgaX* repair template
pgaX -NEST-R	CTCTCATAGTACCCAACCCTGC	
xlnR PM	CTGTGGATCTCCTCGGAGTCGTTTGTCTCGGCCATGAGCCATGCGTTCGGTGCCG CAGAAGCAGCGGCAGAAATCTTGGAGTACGACCCG	*xlnR* point mutation repair template
**Primers for CRISPR/Cas9 sgRNA construction**		
P1-gRNA	CAACCTCCAATCCAATTTGACTCCGCCGAACGTACTG	5F-sgRNA; for amplification of 5' flank region and fusion of sgRNA construct
P2-gRNA	ACTACTCTACCACTATTTGAAAAGCAAAAAAGGAAGGTACAAAAAAGC	3R-sgRNA; for amplification of 3' flank region and fusion of sgRNA construct
P3-*xlnR* N-term	TGCGCTCATTGCATTGGTAGGACGAGCTTACTCGTTTCG	5R-*xlnR* N-terminal region; for amplification of 5' flank region of sgRNA construct
P4-*xlnR* N-term	CTACCAATGCAATGAGCGCAGTTTTAGAGCTAGAAATAGCAAG	3F-*xlnR* N-terminal region; for amplification of 3' flank region of sgRNA construct
P3-*xlnR* PM	CATGGCTCATGGCCGAGACAGACGAGCTTACTCGTTTCG	5R-*xlnR* point mutation; for amplification of 5' flank region of sgRNA construct
P4-*xlnR* PM	TGTCTCGGCCATGAGCCATGGTTTTAGAGCTAGAAATAGCAAG	3F-*xlnR* point mutation; for amplification of 3' flank region of sgRNA construct
P3-*xlnR*	CGGTCTCCTGGCGAGTATGCGACGAGCTTACTCGTTTCG	5R-*xlnR* ; for amplification of 5' flank region of sgRNA construct
P4-*xlnR*	GCATACTCGCCAGGAGACCGGTTTTAGAGCTAGAAATAGCAAG	3F-*xlnR* ; for amplification of 3' flank region of sgRNA construct
P3-*pgaX*	CCTTGAAGATCGGCAGATAGGACGAGCTTACTCGTTTCG	5R-*pgaX* ; for amplification of 5' flank region of sgRNA construct
P4-*pgaX*	CTATCTGCCGATCTTCAAGGGTTTTAGAGCTAGAAATAGCAAG	3F-*pgaX* ; for amplification of 3' flank region of sgRNA construct
P3-*gaaR*	cgtcgcgtccatgccgaaccGACGAGCTTACTCGTTTCG	5R-*gaaR* ; for amplification of 5' flank region of sgRNA construct
P4-*gaaR*	GGTTCGGCATGGACGCGACGGTTTTAGAGCTAGAAATAGCAAG	3F-*gaaR* ; for amplification of 3' flank region of sgRNA construct
**Primers for screening transformant colonies**		
xlnR PM seq-F	TGGATCACACGAGTCCTTCG	F primer for sequencing of *xlnR* point mutation
xlnR PM seq-R	AAGTCCTCTGGGATGCGTCC	R primer for sequencing of *xlnR* point mutation
xlnR N-term seq-F	GTGTCGCCTTAATCTCCTTTCC	F primer for sequencing of *xlnR* N-terminal region
xlnR N-term seq-R	CCTAGCGATGATTGCGAATGC	R primer for sequencing of *xlnR* N-terminal region
pgaX -F	TTGCTTGCCAGTGTTGTAGACC	for screening the presence of *hph* in the correct *pgaX* position
pgaX -R	GGGCGTCGGTTTCCACTATC	

Table S3 Summary of the ANOVA analysis for each enzyme assay and saccharification test.

Code	N. of variables[a]	DF[b]	F-value	*p* -value[c]
**Enzymatic activities**				
BGL	4	23	1640.41	0,0000
BXL	4	23	2495.08	0,0000
EGL	4	23	2495.08	0,0000
XLN	4	23	94.39	0,0000
**Saccharification tests**				
D-gal.acid_SBH_CP	6	23	90.19	0,0000
D-gal.acid_SBH_CPX	6	23	271.14	0,0000
D-gal.acid_CP_CP	6	23	4441.6	0,0000
D-gal.acid_CP_CPX	6	23	1169.35	0,0000
L-arabinose_SBH_CP	6	23	50.88	0,0000
L-arabinose_SBH_CPX	6	23	107.38	0,0000
L-arabinose_CP_CP	6	23	123.34	0,0000
L-arabinose_CP_CPX	6	23	77.61	0,0000
D-xylose_SBH_CP	4	23	6,59	0,0028
D-xylose_SBH_CPX	4	23	711,44	0,0000

[a] Number of variables within each ANOVA analysis

[b] DF: degrees of freedom

[c] *p* -value of the F-test. Statiscal significance is refered for $p < 0.05$.

SBH: soybean hulls

CP: citrus pectin

CPX: citrus pectin + D-xylose

References

Ludwiczak J, Winski A, Szczepaniak K, Alva V, Dunin-Horkawicz S (2019) DeepCoil-a fast and accurate prediction of coiled-coil domains in protein sequences. Bioinformatics 35:2790-2795. https://doi.org/10.1093/bioinformatics/bty1062

Meyer V, Arentshorst M, El-Ghezal A, Drews AC, Kooistra R, van den Hondel CAMJJ, Ram AFJ (2007) Highly efficient gene targeting in the *Aspergillus niger kusA* mutant. J Biotechnol 128:770-775. https://doi.org/10.1016/j.jbiotec.2006.12.021
